# Hereditary pulmonary alveolar proteinosis in a 5-year-old child: Diagnostic insights and therapeutic approach

**DOI:** 10.1016/j.radcr.2025.04.094

**Published:** 2025-05-17

**Authors:** Dhiran Sivasubramanian, Karthick Balasubramanian, Sathwik Sanil, Smrti Aravind, Virushnee Senthilkumar

**Affiliations:** a Department of Cardiology, Children’s hospital of Philadelphia, PA, USA; b Department of Critical Care Medicine, Christian Medical College, Vellore, India; c Institute of Oncology, Sri Ramakrishna Hospital, Coimbatore, India; d Department of General Medicine, Coimbatore Medical College, Coimbatore, India

**Keywords:** Hereditary pulmonary alveolar proteinosis (hPAP), CSF2RA mutation, Whole lung lavage (WLL), Crazy-paving pattern, Bronchoalveolar lavage (BAL), Pediatric respiratory disease

## Abstract

Hereditary pulmonary alveolar proteinosis (hPAP) is a rare disorder caused by mutations in the CSF2RA or CSF2RB genes, leading to impaired surfactant clearance by alveolar macrophages and subsequent respiratory dysfunction. A 5-year-old female with a 2-year history of poor weight gain, fatigue, and intermittent fever was evaluated. Clinical evaluation revealed hypoxemia, while high-resolution computed tomography (HRCT) of the chest showed the characteristic “crazy-paving” pattern suggestive of PAP. Bronchoalveolar lavage (BAL) yielded milky fluid with periodic acid-Schiff (PAS)-positive material, and genetic testing confirmed a homozygous mutation in the CSF2RA gene, consistent with hPAP. The patient underwent therapeutic whole lung lavage (WLL), resulting in significant clinical improvement. This case underscores the challenges of diagnosing pediatric hPAP and the value of integrating imaging, pathology, and genetic testing. While WLL remains the mainstay of treatment, further research is needed to develop targeted therapies.

## Introduction

Pulmonary alveolar proteinosis (PAP) is a rare and potentially fatal form of interstitial lung disease, which is characterized by the accumulation of lipoproteinaceous material, mainly composed of surfactant phospholipid and apoproteins, in the alveoli. This leads to impaired gas exchange and progressive respiratory insufficiency. It results from a disruption in the clearance of surfactant by alveolar macrophages, which normally regulates surfactant homeostasis in the lungs [[1], [2], [3]]. Based on the etiopathogenesis, PAP is classified into three major types: 1) Autoimmune PAP (primary)—the most common form accounting for about 90% of cases, 2) Secondary PAP—occurs as a consequence of underlying conditions such as malignancies, inhalational exposure or immunodeficiencies, which impair macrophage function, 3) Hereditary PAP (hPAP)—a rare genetic form caused by mutations in the CSF2RA or CSF2RB genes, leading to impaired surfactant clearance by alveolar macrophages [1].

Clinically, hPAP often presents in infancy or early childhood with nonspecific respiratory symptoms, including progressive dyspnea, cough, and hypoxemia. Radiologically, high-resolution computed tomography (HRCT) often suggests the diagnosis [1]. But diagnosis requires confirmation through genetic testing, a bronchoalveolar lavage (BAL), or a lung biopsy in select cases [4].

The management of hPAP remains challenging, and it is primarily supportive. Whole lung lavage (WLL) is the mainstay therapy [1,4,5].

We present a case of hereditary pulmonary alveolar proteinosis in a child, aiming to highlight the diagnostic challenges and management considerations associated with hPAP.

## Case report

A 5-year-old female was brought to the outpatient clinic with complaints of poor weight gain and increased fatigue while playing with her peers, symptoms she has been experiencing for the past two years. Her parents also reported intermittent episodes of high-grade fever, occurring every three months and lasting about 10 days, during this period. Additionally, they observed that she was shorter than her peers. She had multiple hospitalizations since the age of 3 for recurrent fevers and pneumonia, which were treated with intravenous antibiotics. She was born to parents who were married consanguineously in the second degree, and her antenatal history was unremarkable. All developmental milestones were achieved on time, and her vaccinations were up to date. Her diet was normal, with adequate protein and calorie intake. There was no family history of any similar illnesses.

**Fig. 1:**
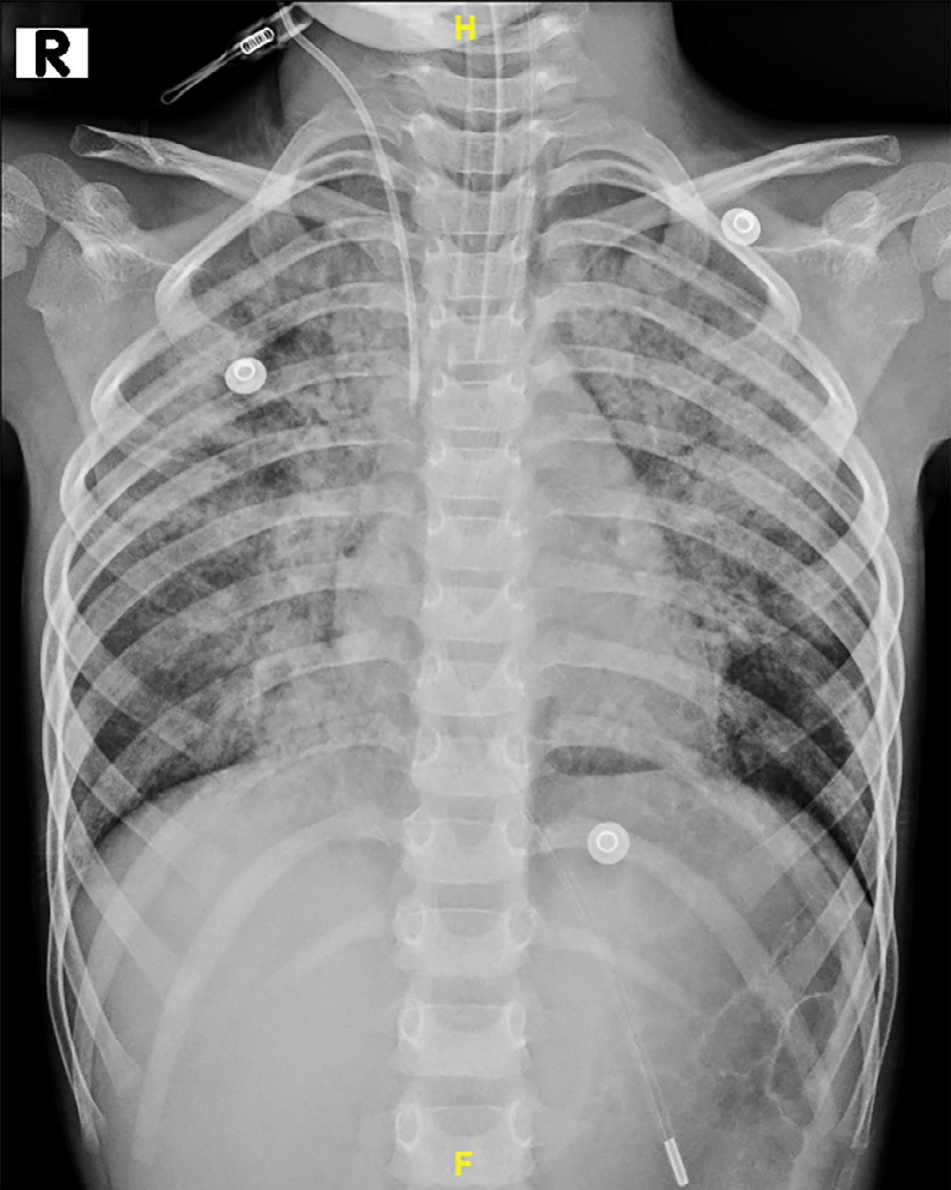
Chest radiograph (X-ray) showing bilateral diffuse nonhomogeneous opacities.

**Fig. 2:**
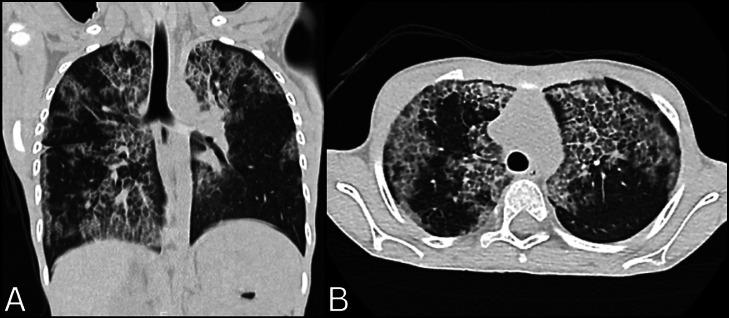
High-resolution computed tomography (HRCT) (A) coronal and (B) axial view of the chest showing large patchy areas of ground glass opacities with interlobular septal thickening involving all lobes of bilateral lung parenchyma, resulting in a crazy-paving pattern.

Upon presentation, her vital signs were as follows: blood pressure of 90/60 mmHg, pulse rate of 100 beats per minute, respiratory rate of 25 breaths per minute, and an oxygen saturation of 95%. The physical examination was normal, with no abnormalities noted in the cardiovascular, respiratory, or central nervous systems. Routine blood tests were within reference range (Table 1). Arterial blood gas analysis showed hypoxemia with a PaO_2_ of 34 mmHg, a PaCO_2_ of 41 mmHg, and a pH of 7.36. A Chest radiograph (X-ray) demonstrated bilateral, diffuse, non-homogeneous opacities (Fig. 1). Followed by a high-resolution computed tomography (HRCT) of the chest that showed large, patchy areas of ground-glass opacities with interlobular septal thickening across all lobes of both lungs, resulting in a classic “crazy-paving” pattern (Fig. 2). Additionally, there was mild involvement of the right upper paratracheal and subcarinal lymph nodes, measuring up to 3 mm. The complete antinuclear antibody (ANA) panel was negative.

**Table 1:** Routine blood investigations.

Investigation	Patient value	Reference value
Hemoglobin (Hb)	14 g/dL	12-15 g/dL
Mean corpuscular volume (MCV)	82.1 fL	80-100 fL
Platelet count	327,000 cells/µL	150,000-450,000 cells/µL
Total white blood cell count (WBC)	5,800 cells/μL	4,500-11,000 cells/μL
Total bilirubin	0.37 mg/dL	0.3-1.2 mg/dL
Direct bilirubin	0.10 mg/dL	0-0.3 mg/dL
Serum albumin	5.0 g/dL	3.5-5 g/dL
Alanine transaminase (ALT)	44 IU/L	5-44 IU/L
Aspartate aminotransferase (AST)	18 IU/L	5-35 IU/L
Alkaline phosphatase (ALP)	315 U/L	273.47-871.44 U/L
Urea	39 mg/dL	7-40 mg/dL
Creatinine	0.34 mg/dL	0.5-1.2 mg/dL
Bicarbonate	21 mmol/L	22-29 mmol/L
Sodium	138 mmol/L	136-145 mmol/L
Potassium	4.3 mmol/L	3.5-5.1 mmol/L

Given the radiological suspicion of PAP, the patient underwent bronchoscopy with bronchoalveolar lavage (BAL) of the right lower lobe. The BAL fluid appeared milky and turbid, becoming increasingly dense with each lavage. Periodic acid-Schiff (PAS) staining of the fluid was positive, with the granular material consistent with alveolar proteinosis. The total white blood cell count in the BAL fluid was 360/mm³, with 28% lymphocytes and 65% macrophages. Granulocyte-macrophage colony-stimulating factor (GM-CSF) autoantibodies were not detected in either the serum or the BAL fluid. Genetic testing revealed a homozygous mutation in the CSF2RA gene, confirming the diagnosis.

**Fig. 3:**
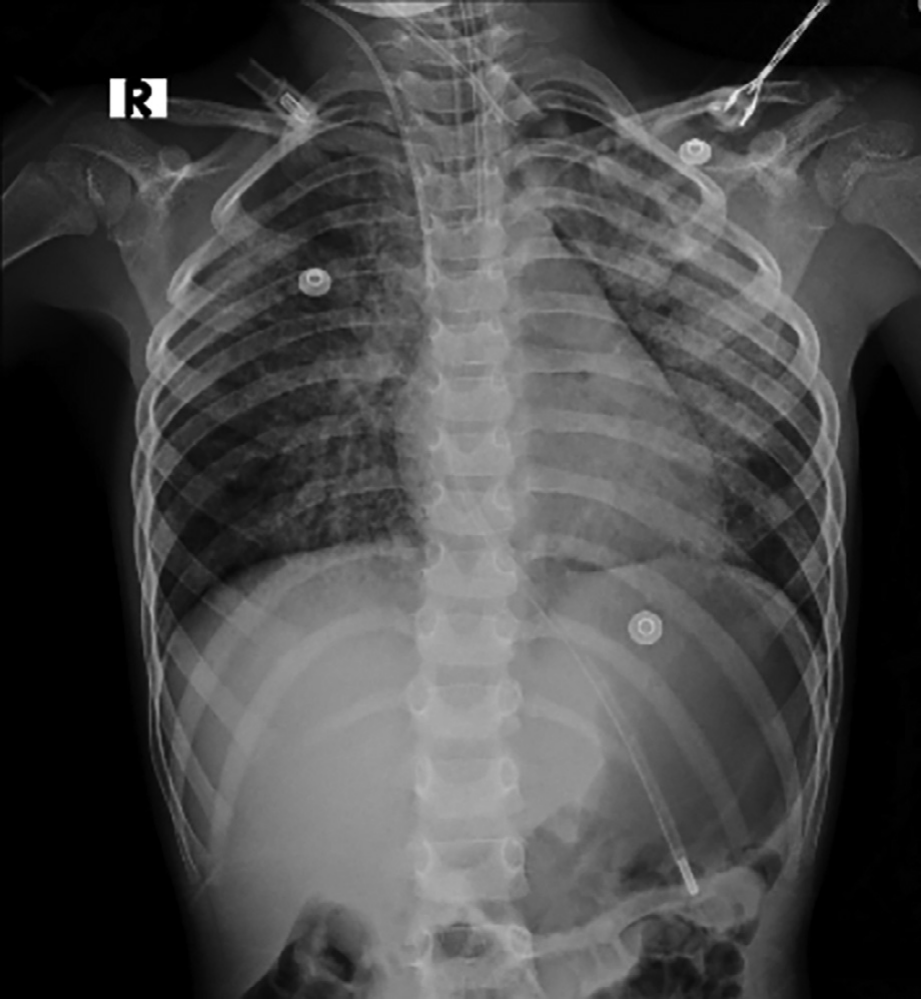
Chest radiograph (X-ray) post right whole lung lavage (WLL) showing reduction of the opacities in the right lung in comparison to the first X-ray in Fig. 1.

The patient underwent therapeutic whole-lung lavage (WLL) under general anesthesia, with successful fluid clearance achieved after 15 cycles. A chest radiograph taken a day after the procedure showed a significant reduction in the opacities over the right lung (Fig. 3) in comparison to (Fig. 1). The procedure was repeated a few more times over the month. At a follow-up visit the next month, she had a significant improvement in her symptoms.

## Discussion

Pulmonary alveolar proteinosis is rare in children, with an estimated incidence of two cases per million under the age of 18 [6]. While the majority of them are autoimmune PAP (primary), hereditary PAP is extremely rare, accounting for less than 6% of these cases [3]. The CSF2RA or CSF2RB genes encode the alpha and beta subunits of the granulocyte-macrophage colony-stimulating factor (GM-CSF) receptor, respectively [1]. These mutations disrupt surfactant homeostasis by impairing the alveolar macrophages' ability to clear surfactant, leading to accumulation in the alveoli and resulting in respiratory dysfunction [2,3].

The patient presented with nonspecific symptoms that had persisted over two years. These symptoms, along with a history of recurrent pneumonia and consanguineous parentage, suggested a possible underlying genetic disorder. Routine blood investigations were largely unremarkable, except for hypoxemia. However, the imaging findings, particularly the HRCT scan, were crucial in establishing the diagnosis. The initial chest radiograph showed diffuse, non-homogeneous opacities—a non-specific finding often seen in various pulmonary pathologies [3]. Although a chest radiograph can serve as an initial screening tool [3], it was the HRCT that revealed the hallmark “crazy-paving” pattern of PAP [1,7]. This pattern is named for the resemblance of ground-glass opacities superimposed with interlobular septal thickening to a pathway paved with irregularly broken pieces of stone [7]. Typically, the opacities are bilateral and diffuse, often involving the perihilar and basal lung regions, as seen in this child. Lymphadenopathy, although less common, can be observed in PAP and is more frequently seen in secondary forms. It may reflect a reactive process or an association with infections due to impaired pulmonary defense mechanisms [8].

The “crazy-paving” pattern can also be seen in other conditions, such as cardiogenic pulmonary edema, acute respiratory distress syndrome (ARDS), pneumocystis pneumonia (PCP), and various neoplastic processes [9,10]. Therefore, the diagnosis must be confirmed with additional tests, including BAL (the gold standard) and genetic studies in hereditary cases. In the appropriate clinical context, particularly in pediatric cases with unresolved respiratory symptoms and growth concerns, this pattern should raise strong suspicion for PAP.

The milky appearance of the BAL fluid, along with positive PAS staining, confirmed our diagnosis of PAP. The absence of GM-CSF autoantibodies excluded the autoimmune form of PAP, while genetic testing identified a homozygous mutation in the CSF2RA gene, confirming the hereditary form [9]. This distinction is crucial for diagnosis and treatment, as recombinant GM-CSF therapy is effective in autoimmune PAP but unlikely to work in hPAP due to the underlying receptor dysfunction [9,11].

The management of hPAP is primarily supportive, with WLL being the cornerstone of therapy. WLL involves the physical removal of accumulated surfactant material from the alveoli, improving gas exchange and respiratory function [12]. However, WLL is not a cure, and patients often require repeated procedures [9,12]. The long-term prognosis of hPAP varies, and some patients may develop progressive respiratory failure despite treatment. In such cases, lung transplantation may be considered [12].

The genetic basis of hPAP raises important considerations for family counseling and screening. In this case, the consanguineous parents increased the likelihood of an autosomal recessive disorder such as hPAP. Genetic counseling should be offered to the family, and siblings should be screened for the CSF2RA mutation, even if they are asymptomatic [13]. Early diagnosis and intervention in affected siblings can improve outcomes and prevent complications.

## Conclusion

This case highlights the diagnostic challenges associated with hPAP in children and emphasizes the importance of a systematic approach to evaluation, including clinical, radiological, histopathological, and genetic assessments. HRCT remains essential in the radiological evaluation of PAP. Recognizing the “crazy-paving” pattern in children with chronic respiratory symptoms should prompt consideration of hereditary PAP, necessitating a comprehensive diagnostic approach involving bronchoscopy and genetic testing. While WLL remains the primary treatment, further research is needed to develop targeted therapies for the underlying genetic defects in hPAP.

## Patient consent

Written informed consent for publication of this case was obtained from the patient.
